# The Louisville Medical School

**Published:** 1850-03

**Authors:** 


### THE WESTERN JOURNAL.

THIRD SERIES.—VOL. V.—No. III.

LOUISVILLE, MARCH 1, 1850.

---

### THE LOUISVILLE MEDICAL SCHOOL.

The medical department of Louisville University has just terminated the most successful, useful and brilliant session of its career. We promised great and important results from the changes made last summer and fall in the Faculty, and we believe that all our predictions have been more than fulfilled. In the department of clinical teaching, the success has been all that could have been asked for. This important branch of teaching, which is so shamefully conducted, or neglected, generally, in the West and South, has received vitality at the hands of Professor Bartlett, and we feel fully justified in saying that no medical class ever received a more valuable course of clinical instruction than the recent class in the medical school of Louisville. Professor Bartlett is eminently adapted to the business of clinical teaching; he is thoroughly acquainted with the pathology of disease; he is proficient in all the enlightened methods of investigation known to physicians; he is a master of diagnosis, and possesses remarkable descriptive powers. His manner is more impressive than that of any clinical teacher we have ever known, and dull, indeed must that student be, who can listen to Prof. Bartlett's clinical teaching without profiting largely by it.

The Hospital, under its present arrangement, is admirably fitted for clinical instruction. The joint committee of the legislature thus speaks of the institution:

"A large number of invalids, male and female, tenanted and occupied the different apartments of the building, and all the offices of "good Samaritanism" seemed to be actively employed by those entrusted with the care and management of the concerns of the hospital.

"Cleanliness and comfort were everywhere visible, and every want of the sick was apparently supplied.

"The rooms of the building are large, comfortable, well lighted, and susceptible of the most excellent ventilation.

"We think too much praise cannot be bestowed upon those whose efforts have so potently contributed to smooth the pillow and soften the bed of the afflicted."

These facts hold out inducements to patients to go into the hospital, and while they are greatly benefitted by the care bestowed upon them, the material for clinical instruction is ample and varied.

Professor Silliman's success has justified the highest anticipations of his warmest admirers. His course of lectures has been received with great favor by the class, and its members are enthusiastic in their expressions of approbation. There is no difference of opinion, among the students, as to the excellence of Professor Silliman's lectures; they all speak in warm terms of the fullness, clearness and impressiveness of his lectures, and of his rare skill as a manipulater and experimenter.

Our friend, Professor Rogers, too, has fully justified the expectations of his friends. Their expectations were very high, but he has fully come up to them. We have never known a young lecturer who seized success with a firmer grasp.

We congratulate the lovers of medical science upon the auspicious circumstances of the Louisville medical school. No institution in the country has stronger claims npon those who wish to obtain a thorough knowledge of medical science in all its departments.

